# Lipid levels in HIV-positive men receiving anti-retroviral therapy are not associated with copy number variation of reverse cholesterol transport pathway genes

**DOI:** 10.1186/s13104-015-1665-z

**Published:** 2015-11-21

**Authors:** Rebecca B. Marino, Lawrence A. Kingsley, Shehnaz K. Hussain, Jay H. Bream, Sudhir Penogonda, Priya Duggal, Jeremy J. Martinson

**Affiliations:** Department of Infectious Diseases and Microbiology, Graduate School of Public Health, University of Pittsburgh, 130 De Soto St, Pittsburgh, PA 15261 USA; Division of Hematology/Oncology, Department of Medicine, Cedars-Sinai Medical Center, 8700 Beverly Blvd, Los Angeles, CA 90048 USA; Bloomberg School of Public Health, Johns Hopkins University, 615 Wolfe St, Baltimore, MD 21205 USA; Feinberg School of Medicine, Northwestern University, 645 N Michigan Avenue, Chicago, IL 60611 USA

**Keywords:** CNV, Copy number variation, Dyslipidemia, HIV-1, Reverse cholesterol transport pathway, Anti-retroviral therapy

## Abstract

**Background:**

The exacerbation of HIV-1 associated dyslipidemia seen in a subset of patients receiving anti-retroviral therapy suggests that genetic factors put these individuals at greater risk of cardiovascular disease. Single nucleotide polymorphisms (SNPs) within genes of and influencing the reverse cholesterol transport (RCT) pathway are associated with lipid levels but little is known regarding their copy number variation (CNV). This form of quantitative genetic variation has the potential to alter the amount of gene product made, thereby also influencing lipid metabolism.

**Results:**

To examine if CNV in RCT pathway genes was associated with altered serum lipid profiles in HIV-positive individuals receiving therapy, we designed a custom multiplex ligation-dependent probe amplification assay to screen 16 RCT genes within a subset of individuals from the Multicenter AIDS Cohort Study who show extreme lipid phenotypes. Verification of CNV was performed using a custom NanoString assay, and the Illumina HT-12 mRNA expression microarray was used to determine the influence of copy number on gene expression. Among the RCT genes, CNV was observed to be extremely rare. The only CNV seen was in the *CETP* gene, which showed a loss of copy in 1 of the 320 samples (0.3 %) in our study. The genes in our study showed little variation in expression between individuals, and the variation seen was not related to any detected CNV.

**Conclusions:**

Whole gene CNV is uncommon in RCT pathway genes, and not a major factor in the development of highly active antiretroviral therapy (HAART) associated dyslipidemia.

**Electronic supplementary material:**

The online version of this article (doi:10.1186/s13104-015-1665-z) contains supplementary material, which is available to authorized users.

## Background

Individuals infected with HIV-1 exhibit changes in serum lipid levels seen as hypercholesterolemia and hypertriglyceridemia [1–4]. Following antiretroviral therapy (ART), lipid levels remain skewed for many patients, as LDL-cholesterol (LDL-C) and triglycerides increase while HDL-cholesterol (HDL-C) remains lowered [1–3, 5–7].

Previous studies have shown that this dyslipidemic profile is associated with greater risk for cardiovascular disease (CVD), myocardial infarction and atherosclerosis in HIV-positive individuals [3, 5, 8–11]. As some in the HIV-1 infected population have begun to reach the age where CVD risk is increased and the affect of HIV infection on this risk in unknown, there is a need to understand the mechanisms behind therapy-associated lipid dysfunction. Many factors likely contribute towards this dyslipidemia, including the different drug components used in ART [12–14] as well as factors innate to the individual. The prevalence of dyslipidemia is high, but not all-inclusive, among the HIV-positive population suggesting that genetic factors potentially have a role [15]. Studies have already illustrated a broad genetic impact on lipids, as lipid levels and CVD risk vary based on ethnic background in HIV uninfected populations [16–18]. We have recently shown that biogeographical ancestry was significantly associated with lipid levels in a cohort of men who have sex with men (MSM), and that European ancestry results in a more atherogenic phenotype even after controlling for HIV and therapy components [19].

Furthermore, several genome-wide association studies (GWAS) have identified single nucleotide polymorphisms (SNPs) associated with CVD risk [20–25], many of which are present in genes involved in cholesterol metabolism and transport. One particularly relevant set of genes is that of the reverse cholesterol transport (RCT) pathway, which directly influences lipid levels. Polymorphisms in genes of this pathway, and in those directly interacting with it, contribute to the variance of lipid levels, and also alter expression levels of some of the genes themselves [15, 26–33]. Recent studies have identified specific mutations located within RCT genes that are associated with altered lipid levels in individuals with HIV-associated atherogenic dyslipidemia. The SNP rs3135506, located in the *APOA5* gene, was associated with increased triglycerides and decreased HDL-C, while a SNP in the *LPL* gene, rs328, was associated with increased levels of HDL-C [34]. Additionally, common SNP genotypes in *APOE* were found to be associated with lipid levels where the E2 allele has a protective effect against dyslipidemia while the E4 is indicative of more risk [35]. Furthermore, expression levels of *LDLR* can be modified by mutations in the proprotein-convertase subtilisin-kexin type 9 gene (*PSCK9)*, ultimately resulting in altered levels of LDL-C [27, 36, 37]. Individuals with loss of function mutations in *PCSK9* show decreased amounts of LDL-C while those with gain of function mutations have increased amounts [26, 33, 38].

In addition to posttranslational protein regulation such as that seen with *PCSK9*, protein levels of RCT gene products could also be influenced by copy number variation (CNV). This type of genetic variation includes duplications, deletions, and inversions of DNA segments greater than 50 bp in size [39–42]. Previous studies on CNV in the *CCL3L1* and *DEFB4* genes illustrate that an increase in transcriptionally available copies of a gene not only results in increased expression levels but also increases in protein levels directly proportional to the number of copies [43–46]. Such variation in one or a few RCT genes has the potential to alter the functionality of this lipid metabolism pathway dramatically, and thereby influence serum HDL and LDL levels. Yet, while there have been a number of studies investigating the association of SNPs within these genes to lipid levels [28–32, 47], little has been documented related to their CNV, apart from reports on rare structural variation in the *LDLR* gene associated with Familial Hypercholesterolaemia [48–51] and the occasional reported variant in *LPL*, *ABCA1*, and *LIPC* [48, 52]. Furthermore, the Database of Genomic Variants, a compilation of structural variation in healthy control sample genomes, contains rare CNV encompassing the RCT genes [53].

Here, we designed a study employing custom Multiplex Ligation-dependent Probe Amplification (MLPA) and NanoString probes to screen for CNV in 16 RCT associated genes in participants from the Multicenter AIDS Cohort Study (MACS), to identify if CNV is present, the degree to which it varies, and whether it has an association with the abnormal lipid metabolism observed in HIV-positive individuals undergoing antiretroviral therapy.

## Results

### Sample demographics

**Fig. 1:**
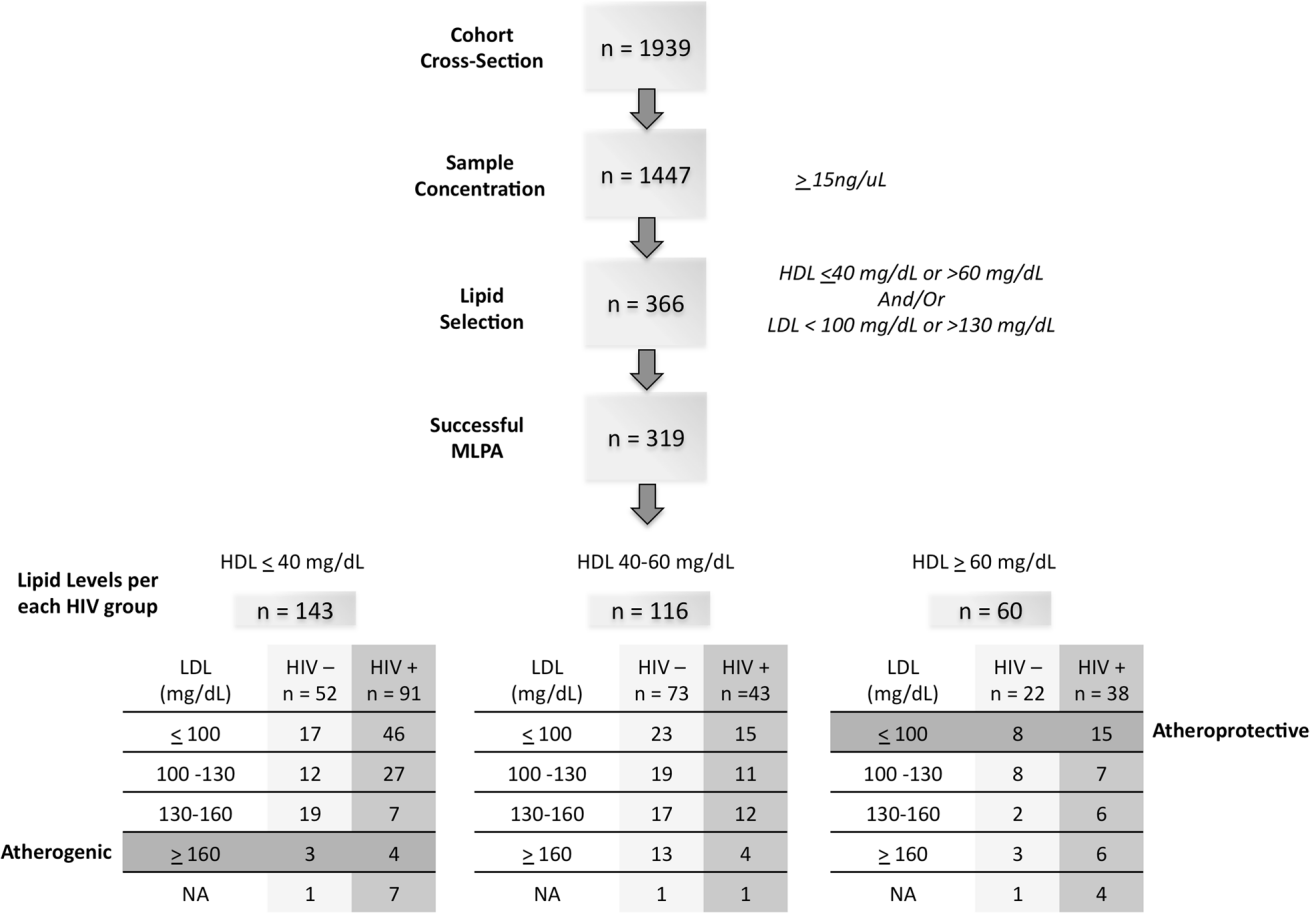
Sample Subset Selection Methodology. Samples were initially selected on the basis of DNA quality. HDL and LDL measurements were then used to identify atheroprotective and atherogenic subgroups, and control samples with HDL and LDL values in the normal range were selected to complete the sample set

Using the 2005 clinic measurements, and the NCEP/ATP III report criteria [54] (HDL-C ≤40 mg/dL or ≥60 mg/dL; LDL-C ≤100 mg/dL or ≥130 mg/dL), 366 suitable MACS participants were identified, of which 319 were successfully analyzed using MLPA (Fig. 1). The demographic data for these 319 samples are summarized in Table 1. We identified 23 samples with an atheroprotective phenotype (HDL-C ≥60 mg/dL and LDL-C ≤100 mg/dL) and 7 samples with an atherogenic phenotype (HDL-C ≤40 mg/dL and LDL-C ≥160 mg/dL). Those with the atherogenic lipid profile had a higher mean body mass index (BMI), plus higher total cholesterol and triglyceride levels when compared to those who had the atheroprotective phenotype. Age among all lipid groups was similar, with a median age of 48 (IQR: 47–49). BMI was higher in the uninfected individuals within each grouping and, with the exception of the atheroprotective group, the mean BMI of most groups ranged from overweight to borderline obese.

**Table 1:** Demographic and descriptive characteristics of study participants

	HDL ≤ 40 mg/dL										HDL = 40–60 mg/dL					
LDL	≤100 mg/dL		100-130 mg/dL		130–160 mg/dL		≥160 mg/dL		LDL Not Measured		≤100 mg/dL		100–130 mg/dL		130–160 mg/dL	
HIV Status	HIV−	HIV+	HIV−	HIV+	HIV−	HIV+	HIV−	HIV+	HIV−	HIV+	HIV−	HIV+	HIV−	HIV+	HIV−	HIV+
n	17	46	12	27	19	7	3	4	1	7	23	15	19	11	17	12
*Median*																
Age (years)	51	48.5	53	48	45	55	40	46.5	56	46	48	46	50	50	48	46
BMI	31.6	25.2	27.8	25.2	28	23.9	27.5	30.2	30.6	23.4	27.1	24.7	26.4	24.8	26.9	24.2
HDL (mg/dL)	34	29.1	34.5	31	36	34	37	35.9	28	28.6	50	48.2	50	48	51	46.7
LDL (mg/dL)	78	78.5	121	115	145	142	172	175	130	–	76	86	112	110	145	140
TCHOL (mg/dL)	153	143	189	188	213	214	241	253	181	182	146	151	185	187	215	225
TRIG (mg/dL)	203	197	205	225	152	156	132	148	114	434	81	102	110	108	101	178
*# on therapy*																
No therapy	–	12	–	9	–	2	–	1	–	2	–	3	–	–	–	2
Mono-therapy	–	–	–	–	–		–	–	–	–	–	–	–	1	–	–
Combination	–	5	–	1	–		–	–	–	–	–	–	–	–	–	–
Potent ART	–	29	–	17	–	5	–	3	–	5	–	12	–	10	–	10
*Therapy adherence*																
100 %	–	10	–	7	–	3	–	2	–	–	–	3	–	4	–	5
95–99 %	–	19	–	8	–	2	–	1	–	5	–	8	–	6	–	4
<75 %	–	3	–	2	–		–	–	–	–	–	1	–	–	–	1
NA	–	14	–	10	–	2	–	1	–	2	–	3	–	1	–	2
*BGA*																
AEA	–	4	3	5	4	–	–	1	–	–	6	3	7	2	1	2
AsEA	1	2	–	–	–	–	–	–	–	1	–	–	–	–	2	1
EA	15	39	8	22	15	7	3	3	1	6	16	12	12	9	14	9
NA	1	1	1	–	–	–	–	–	–	–	1	–	–	–	–	–
Group total	17	46	12	27	19	7	3	4	1	7	23	15	19	11	17	12

	HDL = 40–60 mg/dL				HDL ≥ 60 mg/dL											
LDL	≥160 mg/dL		LDL not measured		≤100 mg/dL		100–130 mg/dL		130–160 mg/dL		≥160 mg/dL		LDL not measured			
HIV Status	HIV−	HIV+	HIV−	HIV+	HIV−	HIV+	HIV−	HIV+	HIV−	HIV+	HIV−	HIV+	HIV−	HIV+		
n	13	4	1	1	8	15	8	7	2	6	3	6	1	4		
*Median*																
Age (years)	50	49	22	52	45	42	46.5	52	53.5	47	59	47	31	45.5		
BMI	27.8	26.2	20.7	27.4	25.7	22.9	24.8	23.9	27.3	22.6	26.6	22.5	22.7	24.7		
HDL (mg/dL)	51	45	54.6	40.6	73.5	76.1	63.3	75.9	64.3	82.4	62.5	68.5	60	71.8		
LDL (mg/dL)	165	175	–	–	92.5	80	119	111	138	144	167	167	130	–		
TCHOL (mg/dL)	245	251	163	176	181	181	203	205	223	249	247	259	224	236		
TRIG (mg/dL)	134	122	–	–	86	103	104	94	101	102	105	98	168	525		
*# on therapy*																
No therapy	–	–	–	–	–	4	–	1	–	2	–	2	–	–		
Mono-therapy	–	–	–	–	–	–	–	–	–	–	–	–	–	–		
Combination	–	–	–	–	–	2	–	–	–	–	–	–	–	–		
Potent ART	–	4	–	1	–	9	–	6	–	4	–	4	–	4		
*Therapy adherence*																
100 %	–	2	–	–	–	5	–	1	–	–	–	3	–	2		
95–99 %	–	2	–	–	–	3	–	4	–	2	–		–	2		
<75 %	–	–	–	1	–	3	–	1	–	2	–	1	–	–		
NA	–	–	–	–	–	4	–	1	–	2	–	2	–	–		
*BGA*																
AEA	3	–	1	–	1	11	3	–	–	4	–	–	–	1		
AsEA	–	–	–	–	1	3	–	–	–	–	–	–	–	1		
EA	10	4	–	1	6	1	5	7	2	2	3	5	1	2		
NA	–	–	–	–	–	–	–	–	–	–	–	1	–	–		
Group total	13	4	1	1	8	15	8	7	2	6	3	6	1	4		

^BMI, body mass index; HDL-C, high density lipoprotein cholesterol; LDL-C, low density lipoprotein cholesterol; TCHOL, total cholesterol; TRIG, triglycerides; monotherapy, single nucleoside reverse transcriptase inhibitor; Combination, two or more nucleoside reverse transcriptase inhibitors; potent ART, two or more nucleoside reverse transcriptase inhibitors with a protease inhibitor or a nonnucleoside reverse transcriptase inhibitor; BGA, biogeographical ancestry; AEA, African/European ancestry; EA, European ancestry; AsEA, Asian European ancestry^

Of the individuals who were HIV-positive during 2005, over 70 % were receiving a type of antiretroviral therapy (Potent ART, combination, or monotherapy defined according to the DHHS/Kaiser panel criteria [55]). Of those who reported therapy use, around three quarters had over 95 % self-reported adherence. We also compared the distribution of Biogeographical Ancestry (BGA), recently determined for these samples [19], for the sample subset. The majority of samples in each group were those of European ancestry, followed by those of mixed African-European ancestry, and a few samples with Asian-European ancestry. However in the HIV-positive groups with high HDL-C (≥60 mg/dL) and in the atheroprotective group, samples with African-European ancestry were in the majority.

### Multiplex ligation dependent probe amplification

As reference samples with known copies of the RCT genes are not available, we identified experimental samples whose normalized peak height for each probe was similar to the sample set mean height. Using these samples as references, the coffalyser.net software calculated the probe ratios for each sample relative to the reference samples. Assuming that the most frequently observed ratio corresponded to two copies per diploid genome, ratios above 0.7 and below 1.3 are considered to be within the normal range of two copies [56]. Anything outside of these arbitrary MRC-Holland derived thresholds was identified as an outlier with potential CNV.

**Fig. 2:**
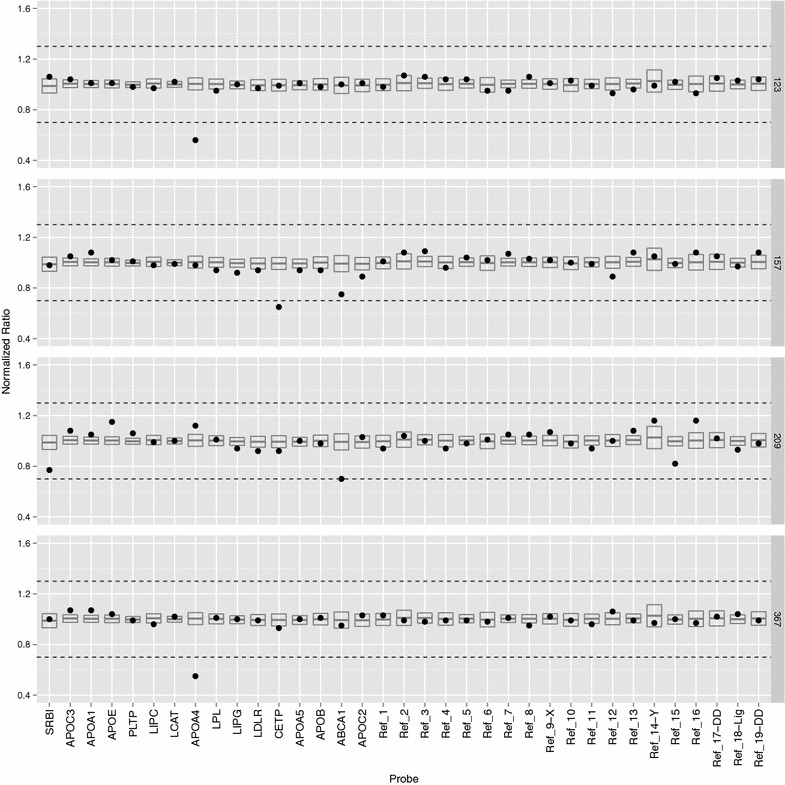
Copy number variation is exceedingly rare for reverse cholesterol transport pathway genes. Copy number ratios are shown for the four individuals that had detectable CNV. Probes representing the RCT genes are on the left of the figure while reference probes (Ref_1–Ref_16), ligation controls (Ref_18), and denaturation controls (Ref_17, Ref_19) are on the right. The dots show the copy number ratios of each probe for each individual. The *box plots* represent the 95 % confidence interval of each probe ratio derived from the entire sample set. Arbitrary thresholds at 1.3 and 0.7 are represented by the *dotted horizontal lines*. Points that fall within these thresholds are considered to have a copy number ratio of 1.0

Of the 16 RCT pathway associated genes screened, only three (*APOA4*, *CETP*, and *ABCA1*) showed any signs of CNV, and in each case the CNV was extremely rare. For each of these genes, a few individuals showed ratios that crossed or were at the lower threshold (Fig. 2). None of the RCT genes had CNV ratios that passed the upper ratio bound of 1.3, suggesting that no sample showed gains in copy number. Table 2 lists normalized ratios of the three genes for samples with losses along with the sample’s 2005 visit HDL-C and LDL-C levels.

**Table 2:** Normalized ratios of reverse cholesterol transport pathway gene CNV probes that showed significant departure from unity

	Lipid levels^a^		Normalized ratios^b^			
Sample	LDL-C, mg/dL	HDL-C, mg/dL	ABCA1	APOA4	CETP	SRBI
123	147 (132.25–162.25)	55 (51–68.3)	1	0.56*	0.99	1.06
157	114 (103–129)	30 (24.8–38.1)	0.75	0.98	0.65*	0.98
209	136.5 (124.25–148.5)	38 (36–40.4)	0.7*	1.12	0.92	0.77
367	69 (49–76)	39.2 (35.8–54.1)	0.95	0.55*	0.93	1.0

^a^The first two columns list median serum HDL-C and LDL-C levels from a minimum of 8 visits for that individual. Within the brackets is the IQR range for those lipid levels

^b^Probes that crossed or were on the 0.7 ratio threshold are indicated with (*)

Two samples (123 and 367) had a loss of *APOA4* copy number (with normalized ratios of 0.56 and 0.55, respectively) while sample 157 had a loss of *CETP* copy number with a ratio of 0.65. Sample 209 had a normalized ratio for *ABCA1* that fell on the 0.7 threshold indicating a possible loss. The standard deviations for each of these outlying probes were relatively small (<±0.05) indicating that the decrease in ratio observed was likely genuine. When MLPA was performed for a second time on these 4 samples, the observations were consistent with the first run. No clear association between any of these losses and lipid levels (Table 2) was apparent, although this observation is not conclusive due to the small number of samples involved.

The interquartile ranges (IQR) for most of the RCT gene probes were narrow (0.04–0.09), and similar to those of the two-copy reference probes. This tight clustering of ratios around the mean of each probe further suggests that CNV is not common in the RCT genes. The only probes with wider IQRs were *ABCA1* and *SRBI*. While their IQRs were slightly broader than the other RCT probes, this spread of the ratio distribution was also seen in the properly functioning reference probes suggesting that this was within the normal range of our experiment (data not shown).

As gold standard referents containing known copy numbers of each RCT gene are not available, and the P300 reference probe set provided by MRC-Holland includes probes with a maximum number of two copies, we developed a quality control assay to ensure that our MLPA protocol was capable of picking up a range of CNV above 2 copies (Additional file 1: Supplementary Methods). Through use of control samples with known amounts of CNV, the MRC-Holland P139 Defensin MLPA assay, and our P300/RCT assay extended to include probes for variants in the *DEFB103A* and *CCR5* genes, we were able to verify that our assay can identify a range of CNV even when the sample mean is used to define a referent (Additional file 2: Figure S2).

To determine whether the rare loss for the three RCT genes identified during MLPA reflected true CNV or problems with probe binding, Sanger sequencing was performed to examine the probe binding site for those individuals who showed losses, plus several control individuals who showed no changes in copy number. We determined that individuals who showed a loss in signal for *APOA4* were heterozygous for a rare intronic SNP rs185210669, located 1 base from the internal ligation site for that probe. This mutant allele fails to bind the left MLPA probe oligo strongly, leading to impaired ligation to the right oligo and decreased MLPA signal. The other genes (*ABCA1* and *CETP*) contained no SNPs within their ligation sites.

### CNV confirmation by NanoString

We confirmed our findings by using a custom NanoString assay to measure CNV of the RCT genes for 267 of the samples analyzed by MLPA. The CNV ratios generated mirrored those seen with MLPA (data not shown). We replicated the loss in copy number of *CETP* in sample 157 (copy number ratio of 0.58) but did not observe the losses for *ABCA1* in sample 209 (1.06) or *APOA4* for both samples 123 and 367 (1.10 and 1.04). As the MLPA-derived ratio for *ABCA1* in sample 209 fell on the threshold value of 0.7, it is likely that this sample does not in fact have a true loss in copies. It is also possible that MLPA probe used for *ABCA1* is picking up a rare small CNV that is not detected by the NanoString probe, as the probes for these assays bind in different regions of the gene. The loss observed in *APOA4* only by MLPA is attributed to the ligation-site SNP identified in the MLPA probe.

### Expression analysis

**Fig. 3:**
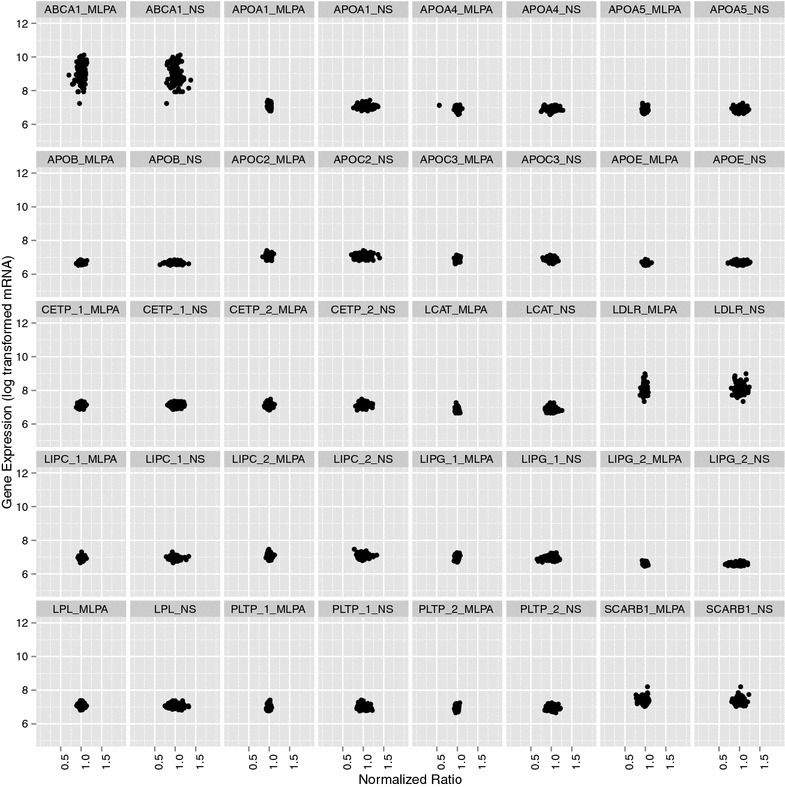
Expression levels of RCT genes. Normalized copy number ratios obtained by MLPA and NanoString are plotted on the *x*-axis while log transformed mRNA expression levels are plotted on the *y*-axis. If expression level data were available for multiple splice variants of the same gene, they were each plotted against their available CNV ratios, with the different variants represented by “_#” following the gene name. Data are shown for the 127 individuals for whom both CNV and expression data were available

We also determined the expression levels of the RCT genes in our study, using data extracted from a whole-genome transcription dataset obtained using the Illumina HT-12 platform. Gene expression levels on 127 samples were compared to both MLPA and NanoString CNV ratios. As expected, comparisons of MLPA- and NanoString-generated CNV ratios to log transformed mRNA expression levels yielded no significant associations (Fig. 3).

## Discussion

The contribution of host genetic variation to the development of the CVD-associated side effects seen in response to antiretroviral therapy is still not fully understood. We have previously studied the roles of Biogeographical Ancestry [19], and of individual SNPs on this, but no studies have been done to date on the impact of quantitative genetic variation such as CNV on this process. To address this, we developed an MLPA assay, and used it to measure CNV in genes within the RCT pathway [28, 29]. As extreme HDL and LDL abnormalities are observed in only a subset of HIV-positive patients receiving anti-retroviral therapy and experiencing dyslipidemia [15], the susceptibility to these severe lipoprotein changes is likely to have a genetic component.

While previous studies have already found an association between sequence variation in genes within, and influencing, the RCT pathway and lipoprotein levels, we theorized that CNV in the RCT pathway could play a role in these extreme lipoprotein phenotypes. A region of duplication encompassing an entire gene and its regulatory regions has the capability to alter expression and protein levels in a manner directly proportional to the amount of copies present. Such a relationship is observed for the *CCL3L1* and *DEFB4* genes, where increases in gene products correspond to copies present for each gene up to a plateau point [43, 46]. Even though this type of variation has the potential to influence lipid metabolism, the available information on whole gene CNV in the genes of the RCT pathway is limited to a few select genes (*LDLR*, *LPL*, *ABCA1*, and *LIPC*) [48, 52].

Data currently available within the Database of Genomic Variants [57] shows a limited amount of rare CNV present for the RCT genes. The documented CNV that is there consists primarily of insertions and deletions within the genes, rather than whole gene variation. Thus, it is unlikely that CNV for these genes is common in the general population, but our strategy here was to combine a population-based screen with a focused investigation of individuals with extreme lipid phenotypes (strongly atheroprotective *vs.* strongly atherogenic). Our hypothesis was that CNV encompassing the full length of a RCT gene would result in an increased or decreased amount of transcribed protein product directly proportional to the amount of copies present, thus impacting serum cholesterol levels. Further, we wished to investigate whether individuals with CNV in the central range would have normal lipid levels while those whose CNV was in the outermost edges of the range would have a dysregulated lipid metabolism leading to the extreme lipid profiles.

Our results in this study identified rare loss variants in 3 of the RCT genes. Out of 267 individuals and 16 genes studied with two different CNV assay procedures, *CETP* showed a loss in a single individual, and two genes (*ABCA1* and *APOA4*) showed apparent copy number losses with MLPA. The apparent loss of copy number seen for *APOA4* was determined to be due to a SNP in the ligation site of its MLPA probe while the apparent loss seen at *ABCA1* is suspected to be not genuine. The small standard deviation seen, along with the reproducibility of the significant loss of signal during additional MLPA runs, indicates that the loss for the *CETP* probe was valid. Coupled with the tight clustering around the normalized ratio of 1.0 for the non-outlying points of all of the RCT probes, these results strongly suggest that whole gene CNV is not present in the RCT genes at anything above very low levels, and is therefore not likely to be a major influence on lipid levels in either the normal population or those infected with HIV and receiving antiretroviral therapy.

These findings are consistent with previous reports of limited structural variation in the RCT genes, as presented in the Database of Genomic Variants [53, 57]. Within this database, deletions that included whole genes were observed for *CETP*. The *ABCA1* gene was observed to have a wide variety of insertions and deletions within its bounds, including five losses in the region of our MLPA probe, although none of them encompass the entire gene. All of these reported variants were extremely rare, with only a few individuals having the variant in studies containing several thousand participants. For those with higher frequencies, the study sizes were too small to conclude that a common variant was observed.

We also compared the ratios obtained with both CNV assays to gene expression data available for a subset of our samples. None of the genes for which we had available CNV data showed variation in expression level. This further suggests that it is unlikely that significant CNV is present in these genes that might affect expression.

### Limitations

Our study was designed to detect any gene-specific CNV in members of the RCT pathway that may impact levels of gene expression and subsequent enzymatic activity that therefore affect lipid levels and lead to dyslipidemia in members of our study cohort. Our study does have some limitations. As no prior survey of CNV in these genes had been attempted, the frequency of such variants was unknown and our study was exploratory. As such, it had limited statistical power to assess the impact of CNV between the atherogenic and atheroprotective groups. Also, the sizes of the atheroprotective (n = 23) and atheroprotective (n = 7) groups are not evenly matched, which would preclude more advanced statistical analysis were we to have found CNV in our samples, but the overall sample size of 319 individuals successfully typed is large enough to have detected any common variants and we can conclude that CNV of these genes is rare or absent. As we only designed one CNV probe set per gene it is also possible that variation within a gene could have been missed—for example, the *LPA* gene contains many kringle repeats that were not a focus of our study.

Our study was also limited by the type of clinical data available. In each biannual visit, serum cholesterol and triglyceride measurements are obtained from our study participants, and were used to define our atherogenic and atheroprotective groups. Other measurements, such as lipoprotein particle size and number, and HDL efflux capacity, have been shown to be stronger predictors of cardiovascular disease risk [58, 59], and would have been collected on subsequent visits had our initial study shown a role for CNV in this pathway. Our samples were also collected prior to the widespread use of the 2013 ACC/AHA guideline, and we therefore use the older NCEP ATP III panel, but our data are based on a sufficiently large sample size to overcome this issue.

## Conclusions

In this study we were able to develop a MLPA assay capable of detecting CNV when it is present. Using this assay we have illustrated that whole gene CNV is present only at very low levels in the RCT genes, and is not a major factor in the development of HAART-associated dyslipidemia. Thus, other host genetic influences exist and need to be identified before we are able to understand fully the ways in which host, viral, and therapeutic environmental factors interact to determine the outcome of antiretroviral therapy in HIV-positive individuals.

## Methods

### Samples

Experimental samples were obtained from the Multicenter AIDS Cohort Study (MACS). The MACS is a multicenter (Baltimore, MD; Chicago, IL; Pittsburgh, PA; and Los Angeles, CA) ongoing prospective study, founded in 1984, of the natural and treated histories of HIV-1 infection in men who have sex with men. Participants attend clinics bi-annually for a physical exam and sample collection, and complete extensive questionnaires about their medical history, behavior changes, and overall quality of life. All samples were obtained from volunteer participants who had read and agreed to the consent policy implemented by the MACS for the protection of human subjects, and approved by the Institutional Review Board (IRB) at each MACS site (The University of Pittsburgh Institutional Review Board; The Johns Hopkins University Institutional Review Board; Northwestern University Institutional Review Board; and The University of California, Los Angeles Medical Institutional Review Board).

Samples (n = 366) were identified on the basis of serum lipid measures obtained in their 2005 visit. We used the Third Report of the National Cholesterol Education Program (NCEP) Expert Panel on Detection, Evaluation, and Treatment of High Blood Cholesterol in Adults (Adult Treatment Panel III) [54] to identify lipid levels associated with higher risk of heart disease (HDL-C <40 mg/dL and/or LDL-C >130 mg/dL) or with lower risk (HDL-C >60 mg/dL and/or LDL-C <100 mg/dL) [60]. DNA was extracted from frozen peripheral blood mononuclear cell pellets using the Qiagen QIAamp DNA Blood Mini Kit, following the Blood and Body Fluid Spin Protocol. DNAs were stored at −20 °C.

We also obtained 4 DNA control samples (NA07048, NA10846, NA10861, and NS12911) from the Coriell Institute cell repository. Three of these individuals served as reference samples, as the CNV of their *DEFB103A* gene was already known [61–63]. We also used 5 DNA samples obtained from volunteer donors in our laboratory as additional controls.

### Copy number variation detection by multiplex ligation-dependent probe amplification (MLPA)

MLPA was performed using the MRC-Holland SALSA MLPA P300 Human DNA Reference-2 (Version: A1-0410) probemix kit, in conjunction with 16 custom probes for RCT pathway genes (Table 3). Custom probes were designed following the manufacturer’s criteria (Synthetic Probe Design v10-update 04-02-2009) utilizing the human genome 18 reference assembly and the Stonybrook MLPA design browser (http://bioinform.arcan.stonybrook.edu/mlpa2/cgi-bin/mlpa.cgi) [64, 65]. BLAST searches were used to verify probe specificity prior to synthesis (Integrated DNA Technologies, Coralville, IA, USA). Additional 5′phosphorylation of the right-hand probe oligo was performed in our laboratory (Additional file 2: Table S1).

**Table 3:** Reverse Cholesterol Transport (RCT) pathway genes selected for analysis

Gene name	Symbol	Chromo-some	Function	References
Scavenger receptor class B, member 1	SRBI	12	Plasma membrane receptor for HDL that mediates transfer of cholesterol to and from HDL	[33]
Apolipoprotein C-III	APOC3	11	Very low density lipoprotein that inhibits lipoprotein lipase and hepatic lipase delaying triglyceride-rich particle catabolism	[32]
Apolipoprotein A-I	APOA1	11	Major protein component of HDL and a cofactor of LCAT. Defects in APOA1 results in HDL deficiencies	[32, 33]
Apolipoprotein E	APOE	19	Main apoprotein of chylomicron and essential for catabolism of triglyceride-rich lipoprotein constituents	[32, 33]
Phospholipid Transfer Protein	PLTP	20	Lipid transfer protein that transfers phospholipids from triglyceride-rich lipoproteins to HDL	[33]
Hepatic Lipase	LIPC	15	Triglyceride hydrolase and ligand/bridging factor for receptor mediated lipoprotein uptake	[32, 33]
Lecithin-cholesterol Acyltransferase	LCAT	16	Extracellular cholesterol esterifying enzyme that esterifies cholesterol for transport	[32, 33]
Apolipoprotein A-IV	APOA4	11	Potent activator of lecithin-cholesterol acyltransferase	[32]
Lipoprotein Lipase	LPL	8	Triglyceride hydrolase and ligand/bridging factor for receptor mediated lipoprotein uptake	[32, 33]
Endothelial Lipase	LIPG	18	Regulates circulating levels of HDL and acts has phospholipase activity	[33]
Low Density Lipoprotein Receptor	LDLR	19	Cell surface protein involved in receptor-mediated endocytosis of LDL	[32, 33]
Cholesteryl ester transfer protein	CETP	16	Transfers cholesteryl esters between lipoproteins	[32, 33]
Apolipoprotein A-V	APOA5	11	Component of high density lipoprotein	[32]
Apolipoprotein B	APOB	2	Main apolipoprotein of chylomicrons and low density lipoproteins	[32]
ATP-binding cassette, sub-family A, member 1	ABCA1	9	Membrane associated protein that functions as a cholesterol efflux pump in the cellular lipid removal pathway	[32, 33]
Apolipoprotein C-II	APOC2	19	Plasma lipid-binding protein that activates lipoprotein lipase	[32]

MLPA reactions were performed following the standard 1 tube protocol (MDP-v001, update 17-06-2011) with an 18–19 h hybridization and amplification step using the new universal primers (released in June 2011). Fragment separation was performed at the University of Pittsburgh Genomics and Proteomics Core Laboratories.

### Analysis of MLPA data

The Coffalyser.net software (http://wiki.coffalyser.net) was used to perform fragment and comparative analysis. Initially, no reference samples were indicated, and samples with average signal across all probes were selected as references for each of the four 96 sample runs. Fragment analysis was then performed for a second time with default settings. Samples with poor reference probe quality and reproducibility were removed before comparative analysis was performed for a second time. Final ratios and standard deviations were analyzed with the R statistical software package [66] and the following modules: ggplot2 [67], reshape [68], and gridExtra [69].

### Copy number calling

For probes that lacked reference samples with known copy number levels, discrete copies were not calculated. Instead, the default ratio thresholds (0.7, 1.3), defined by MRC-Holland and based on a 2-copy reference sample (MLPA Results Interpretation—V02.2;11-02-2010) [70], were used to identify individuals who exhibited a gain (>1.3) or loss (<0.7) of copy number. The interquartile range (IQR) of each probe was also compared to that of the reference probes to identify experimental probes with potential CNV that did not cross the default threshold. If a reference sample with a known discrete copy number was available for a given probe, the discrete copy number of the experimental samples was determined by cluster analysis of raw copy number calls [71] (Additional file 1: Supplementary Methods; Additional file 2: Figure S1 and Table S2).

### CNV confirmation assay

A NanoString nCounter custom CNV CodeSet was designed containing the 16 RCT genes analyzed in our MLPA assay, and used to type 267 of the experimental samples previously typed by MLPA. DNA was processed and analyzed using a NanoString Technologies nCounter system at the University of Pittsburgh Genomics and Proteomics Core Laboratories. The NanoString nSolver Analysis Software (v1.1) was used to generate normalized ratios from raw counts, with 34 of the original MLPA samples set as RCT references.

### Gene expression analysis

Gene expression data were available for 127 of the samples studied by MLPA and NanoString. These were obtained from whole blood collected into PAXgene tubes, from which RNA was extracted using the PAXgene Blood RNA Kit IVD. Samples were quantified, processed, and analyzed using the Illumina Human HT-12 v.4 whole-genome expression array at the University of Pittsburgh Genomics and Proteomics Core Laboratories. Raw data was exported from Illumina Genome Studio and further data analysis was performed using the Bioconductor R modules lumi [72] and ArrayQualityMetrics [73].

### Availability of supporting data

Data are held by the Center for Analysis and Management of the Multicenter AIDS Cohort Study (CAMACS). For access to the MACS data, please complete the collaboration concept sheet and identify the article for which the data were used. This form and instructions may be found at: http://statepi.jhsph.edu/macs/forms.html.

## Additional files

10.1186/s13104-015-1665-z  Supplementary Methods. File Format: PDF.
10.1186/s13104-015-1665-z Supplemental Figures and Tables. **Figure S1.** Reproducible Typing of Rounded Whole Copy Number Calls depends on Distribution of Raw Copy Numbers around Whole Integer Values. **Figure S2.** Probes and reference samples demonstrating a range of CNV further Illustrate the rare nature of CNV in the RCT genes. **Table S1.** Custom MLPA Probe Specifics. **Table S2.** Accuracy of Copy Number Calls is Dependent on Method of Calling. File Format: PDF.

## Abbreviations

ACC/AHA: American College of Cardiology/American Heart Association
ART: antiretroviral therapy
BGA: biogeographical ancestry
CNV: copy number variation
CVD: cardiovascular disease
DNA: deoxyribonucleic acid
GWAS: Genome-Wide Association Study
HDL-C: high density lipoprotein cholesterol
HIV: human immunodeficiency virus
LDL-C: low density lipoprotein cholesterol
MACS: Multicenter AIDS Cohort Study
MLPA: multiplex ligation-dependent probe amplification
MSM: men who have sex with men
NCEP/ATP III: national cholesterol education program/adult treatment panel, third report
RCT: reverse cholesterol transport
SNP: single nucleotide polymorphism
