# Supplementary material for: Lipid levels in HIV-positive men receiving anti-retroviral therapy are not associated with copy number variation of reverse cholesterol transport pathway genes
Source: BMC Res Notes. 2015 Nov 21;8:697. doi: 10.1186/s13104-015-1665-z (PMC4654814; doi:10.1186/s13104-015-1665-z)
Supplement: Supplementary file 1 — 10.1186/s13104-015-1665-z Supplementary Methods. File Format: PDF. [file 13104_2015_1665_MOESM1_ESM.pdf]

Supplementary Methods

MLPA Stonybrook Probe Design Conditions

The Stonybrook MLPA design browser (http://bioinform.arcan.stonybrook.edu/mlpa2/cgi-bin/mlpa.cgi)[1,2] was used with the following conditions: (Hyb Temp = 60°C; Min Tm = 70°C; [Na+] = 0.35M; [Mg2+] = 0; Min delta G = 0 kcal/mol; Protocol = Electrophoresis: stuffer (JS98_v1)) to identify all suitable probes for each gene of the custom set. Custom RCT gene probe sequences selected for use in MLPA are available upon request.

MLPA Fragment Separation Conditions

Fragment separation was performed at the University of Pittsburgh Genomics and Proteomics Core Laboratories, using an ABI 3730xl Genome Analyzer with the following conditions: 1.6 kVolts injection voltage; 25 kVolt run voltage; 50cm capillary; 10 sec injection time; POP7 column; LIZ labeled GS-500 size marker standard.

Positive Control Copy Number Assay

To ensure our MLPA conditions were able to detect a range of CNV when a gold standard CNV reference sample is not available, we genotyped the defensin B103A gene *DEFB103A* and the chemokine receptor gene *CCR5* in a subset of our experimental samples, and in samples from the Coriell repository that have known genotypes for these genes (NA07048 – *DEFB103A* 4 copies/*CCR5* wt/Δ32, NA10846 – *DEFB103A* 5 copies/*CCR5* wt/wt, NA10861 – *DEFB103A* 3 copies/*CCR5* wt/wt). The MRC-Holland P139 Defensin probemix set was selected as a positive control to type the defensin cluster based on its previous use to type CNV in these samples[3-5]. In

addition to the predesigned defensin MLPA kit, we took our original P300/custom RCT probe mix and exchanged 3 of the lipid probes (*APOC2*, *APOA1*, and *APOE*) for a *DEFB103A* probe and another two custom probes designed to detect the full and Δ32 deletion forms of CCR5[6]. The *DEFB103A* probe used was a shortened form of the 04389-L03745 probe from the MRC-Holland P139 probemix, as the original probe length would have conflicted with another reference probe in the P300. The *CCR5* probes consisted of one left hand probe oligo that stopped at the site of the Δ32 deletion and two right hand oligos that were specific to either the wild type sequence or the sequence directly following the Δ32 deletion. With these probes, a wt/wt homozygote has two copies of the wt probe target and zero copies of the Δ32 target, a Δ32/Δ32 homozygote has zero copies of the wt target and two copies of the Δ32 target, and a wt/Δ32 heterozygote has one copy of each probe target.

Copy number calling by clustering

For probes where a reference sample containing a known amount of copies was available, copy number was initially called by multiplying the ratio with the number of copies in the reference then rounding to get a discrete copy. As this yielded inconsistent results for the *DEFB103A* MLPA probes (Additional file 2, Table S2), we typed copy number by applying a *k*-means clustering algorithm to the raw copy number calls[7] (Additional file 2, Figure S1). Additional reference samples with differing copies of the gene of interest served to verify group calls when they clustered with their expected group.

References

1 Zhi J: **MAPD: a probe design suite for multiplex ligation-dependent probe amplification assays**. *BMC Res Notes* 2010, **3**:137–137.

2 Zhi J, Hatchwell E. Human: **MLPA Probe Design (H-MAPD): a probe design tool for both electrophoresis-based and bead-coupled human multiplex ligation-dependent probe amplification assays**. *BMC Genomics 2008*, **9**:407.

3 Fode P, Jespersgaard C, Hardwick RJ, Bogle H, Theisen M, Dodoo D, et al: **Determination of beta-defensin genomic copy number in different populations: a comparison of three methods**. *PLoS ONE* 2011, **6**:e16768.

4 Groth M, Szafranski K, Taudien S, Huse K, Mueller O, Rosenstiel P, et al: **High-resolution mapping of the 8p23.1 beta-defensin cluster reveals strictly concordant copy number variation of all genes**. *Hum Mutat* 2008, **29**:1247–1254.

5 Armour JAL, Palla R, Zeeuwen PLJM, Heijer MD, Schalkwijk J, Hollox EJ: **Accurate, high-throughput typing of copy number variation using paralogue ratios from dispersed repeats**. *Nucleic Acids Research* 2007, **35**:e19–e19.

6 Martinson JJ, Chapman NH, Rees DC, Liu YT, Clegg JB: **Global distribution of the CCR5 gene 32-basepair deletion**. *Nature Genetics* 1997, **16**:100–103.

7 Field SF, Howson JMM, Maier LM, Walker S, Walker NM, Smyth DJ, et al: **Experimental aspects of copy number variant assays at CCL3L1**. *Nat Med* 2009, **15**:1115–1117.
