# Supplementary material for: Lipid levels in HIV-positive men receiving anti-retroviral therapy are not associated with copy number variation of reverse cholesterol transport pathway genes
Source: BMC Res Notes. 2015 Nov 21;8:697. doi: 10.1186/s13104-015-1665-z (PMC4654814; doi:10.1186/s13104-015-1665-z)
Supplement: Supplementary file 2 — 10.1186/s13104-015-1665-z Supplemental Figures and Tables. Figure S1. Reproducible Typing of Rounded Whole Copy Number Calls depends on Distribution of Raw Copy Numbers around Whole Integer Values. Figure S2. Probes and reference samples demonstrating a range of CNV further Illustrate the rare nature of CNV in the RCT genes. Table S1. Custom MLPA Probe Specifics. Table S2. Accuracy of Copy Number Calls is Dependent on Method of Calling. File Format: PDF. [file 13104_2015_1665_MOESM2_ESM.pdf]

Figure S1. Reproducible Typing of Rounded Whole Copy Number Calls depends on Distribution of Raw Copy Numbers around Whole Integer Values.

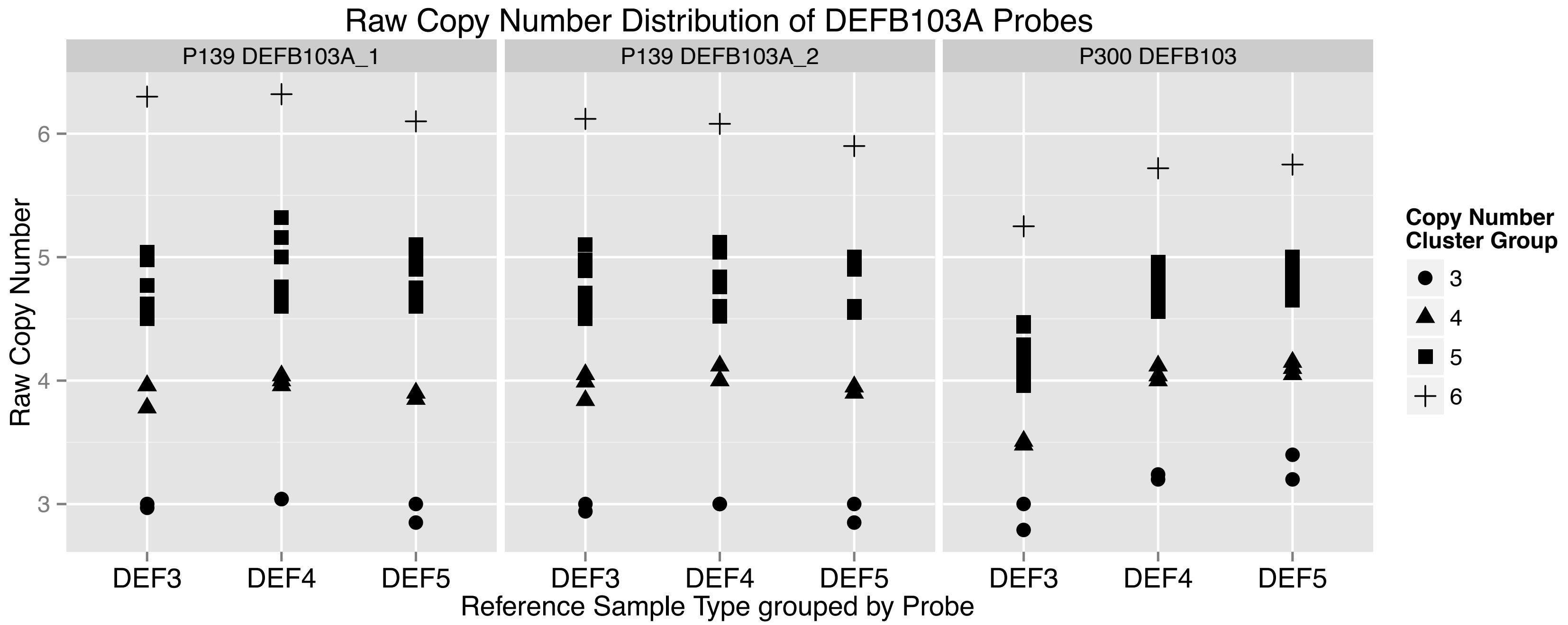

The P300/RCT MLPA assay was extended to include the shorter *DEFB103A* probe from the P139 Defensin assay. Both assays were run simultaneously on 14 samples, including 3 Coriell samples previously typed for *DEFB103A*. Each Coriell sample was set as a reference in separate analyses and the resulting ratios were converted to raw copy numbers by multiplying them with the known number of copies in the reference. The column titles indicate each of the 3 *DEFB103A* probes analyzed while the shape of each point represents the copy number called by *k*-means clustering. Most copy number groups clustered around a whole integer value, with the exception of the 5-copy group. This group skewed towards the 4-copy group, and at times a few samples crossed the halfway point between the two groups resulting in incorrect copy number calls when raw copies were

simply rounded to whole integers. Use of a *k*-means clustering algorithm allowed correct calls each time. All of the raw copy number calls seen for the *DEFB103A* probe in the 3-copy reference sample using the P300/RCT probe set were lower than expected. The run for this sample was of poor quality and therefore ratios generated using it had the potential to fluctuate. Even so, cluster analysis still resulted in proper calls.

Figure S2. Probes and reference samples demonstrating a range of CNV further Illustrate the rare nature of CNV in the RCT genes.

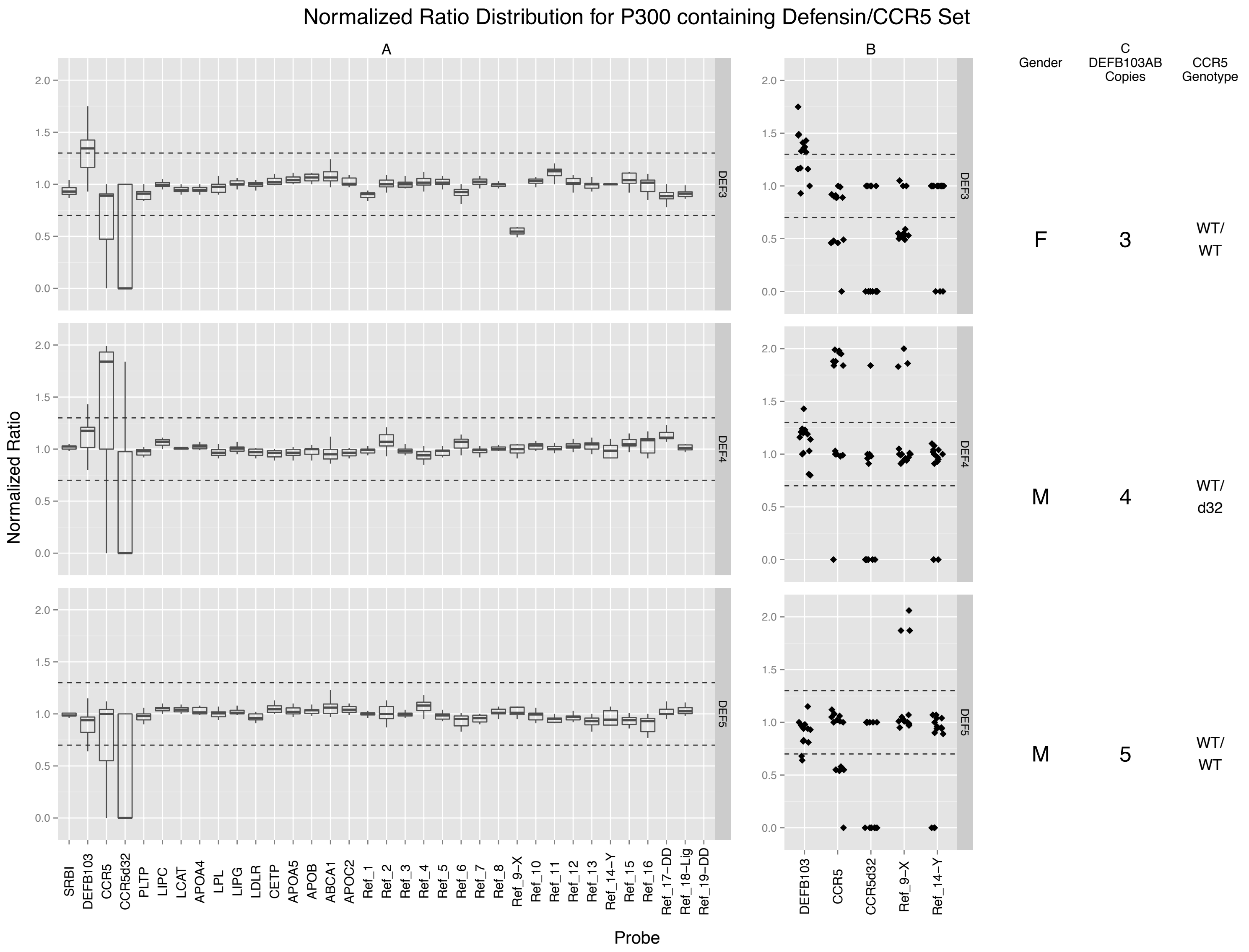

The P300/RCT MLPA assay used in our study was extended to include control probes capable of detecting CNV known to be present in our samples. One probe was designed to detect the *DEFB103A* gene, known to show widespread CNV in humans. Probes were also designed to detect the wild-type and the Δ32 forms of the *CCR5* gene. By using these probes, together with reference samples whose CNV for them was known, we could determine the ability of the default ratio thresholds to detect CNV when the number of copies in the reference sample is unknown. We studied this in a subgroup of 14 individuals (11 males and 3 females; 5 references of known CNV and 9 samples chosen at random from the MACS study set). We set each of our 3 Coriell reference samples as the referent in separate analyses and used the remaining samples to validate the copy number groups identified.

Column A shows the ratios obtained from each analysis using the entire probe set while column B contains the subset of probes that show CNV. Each row in the figure represents the results from each separate analysis using a different reference sample of known CNV and *CCR5* genotype. The *CCR5* genotype, *DEFB103A* copy number, and gender of the referent is shown in column C. Arbitrary thresholds at 1.3 and 0.7 are represented by the dotted horizontal lines. Points that fall inside these thresholds are considered to have the same copy number as the reference sample when a 2 copy reference sample is used. Each dot represents an individual person, the hinges of the box and whisker plots indicate the first and third quartiles of the observed range, and each whisker extends to the furthest value from the median that is within 1.5 * IQR of the hinge.

The referent for the analyses in the top row is a female who is homozygous for the wild-type (wt) allele of the *CCR5* gene and has a validated *DEFB103A* copy number of 3. In this case, the presence of one single additional *DEFB103A* copy in a test sample is sufficient to cross the threshold line and be detectable as a copy number variant. In fact, the 4-copy and 5-copy samples appear distinct from each other. This referent has two copies of the wt allele of *CCR5* and no copies of the Δ32 allele: thus, with the wt allele probe the samples that are also homozygous wt/wt have a copy ratio of 1, the samples with the heterozygous wt/Δ32 genotype have one copy of the wt allele, giving a *CCR5* wt probe copy ratio of 0.5, and the sample that is homozygous Δ32/Δ32 has no copies of the wt allele and gives a wt copy ratio of zero. The ratios obtained with the Δ32 probe show the errors that occur when the referent does not contain any copies of a probe target. In this case, the algorithm cannot discriminate between samples with one copy of the target present and samples with more than one copy present. All of the homozygous wt/wt samples here give a copy ratio of zero, as they do not contain the target for the Δ32 probe, but both the heterozygous wt/Δ32 and homozygous Δ32/Δ32 samples give ratios of 1. Lastly, this referent is female, and therefore has two copies of the X chromosome control probe, and zero copies of the Y chromosome control probe. The male samples thus give a ratio of 0.5 with the X chromosome probe and the female samples give a ratio of 1. With the Y chromosome probe, the females have a probe ratio of zero and the males have a ratio of 1.

The referent for the analysis in the middle row is a male, with 4 copies of *DEFB103A*, who is heterozygous wt/Δ32 at the *CCR5* gene. In this case, both the 3-copy and 4-copy *DEFB103A* samples fall within the threshold lines and cannot be discriminated from each other, but the ratio seen with a 5-copy sample is greater than the threshold and can be identified. The referent has

one copy each of the wt and Δ32 alleles of *CCR5*, thus the *CCR5* genotypes of the samples can easily be determined: with the wt probe, wt/wt homozygotes have a ratio of 2, wt/Δ32 heterozygotes have a ratio of 1, and the Δ32/Δ32 homozygote has a ratio of 0. The reverse relationship is seen with the Δ32 probe. The referent is male, and has one copy each of the X and Y chromosome probe targets. Male samples have one copy of the X chromosome target whereas females have two, and males have one copy of the Y chromosome target and females have zero.

The referent for the analysis in the bottom row is a male, with 5 copies of *DEFB104A*, who is homozygous for the wt allele of *CCR5*. In this case, both the 4-copy and 5-copy *DEFB103A* samples fall within the thresholds and cannot be discriminated from each other, but the 3-copy samples have a probe ratio below the threshold. As this referent lacks the target for the Δ32 probe, the same erroneous ratios are seen as in the top row for this probe, but as it has two copies of the wt probe the wt/wt, wt/Δ32, and Δ32/Δ32 can once again be identified. The sample is a male and therefore has one copy each of the X and Y control probes, allowing the sex of the samples to be identified as in the row above.

These reference samples and probes show that our assay is highly sensitive to detecting CNV when the referent has one or two copies of probe target, and is still sensitive when the referent has three copies. Sensitivity begins to decline when the referent has more copies, but samples that differ from the referent by two or more copies can still be distinguished. Also, the spread of the ratio distributions for the *DEFB103A* probe with true CNV was noticeably larger than that of the 2-copy reference probes and our RCT probes. The IQR did decrease as the copy number of the referent increased but it still remained larger than that

of the 2 copy genes even when the largest *DEFB103A* copy number referent was used. These results suggest that our RCT gene probes have accurately detected the CNV present in our samples, but that this CNV is limited in scope.

Table S1. Custom MLPA Probe Specifics

Oligo Size (nt)	Sequence Name	Sequence	% GC	Tm °C [50mM NaCl]	5' Phosphoryl-ation
46	SRBI_LPO	GGG TTC CCT AAG GGT TGG AAG TGG CCG TCT TGG GCT GGG CGT GTC T	63	74	N/A
50	SRBI_RPO	TCC TGC CTT CAC ACC ACT CGG CCC CAA TCT AGA TTG GAT CTT GCT GGC AC	56	72	In lab
48	APOC3_LPO	GGG TTC CCT AAG GGT TGG AGA AGC ACG CCA CCA AGA CCG CCA AGG ATG	60	73	N/A
52	APOC3_RPO	CAC TGA GCA GCG TGC AGG AGT CCC AGG TGT CTA GAT TGG ATC TTG CTG GCA C	58	72	In lab
50	APOA1_LPO	GGG TTC CCT AAG GGT TGG AGG CGG GGC AGG GGT GTT GGT TGA GAG TGT AC	62	73	N/A
54	APOA1_RPO	/5Phos/TGG AAA TGC TAG GCC ACT GCA CCT CCG CGG ATC TAG ATT GGA TCT TGC TGG CAC	56	72	IDT
54	APOE_LPO	GGG TTC CCT AAG GGT TGG ACA GGA AGA TGA AGG TTC TGT GGG CTG CGT TGC TGG	57	72	N/A
58	APOE_RPO	TCA CAT TCC TGG CAG GTA TGG GGG CGG GGC TTG CTT CTA GAT TGG ATC TTG CTG GCA C	57	73	In lab
56	PLTP_LPO	GGG TTC CCT AAG GGT TGG AGA GTA GGA ATG CAG AGG GCG GAA GGG AGG GCA TCA GT	59	73	N/A
60	PLTP_RPO	AAG CCG ATG GAT GTG GGG ATG CTC AGA GTG GGT TTG ATC TAG ATT GGA TCT TGC TGG CAC	52	71	In lab

58	LIPC_LPO	GGG TTC CCT AAG GGT TGG ATC GGA GGC AGG TCC AGA GAC TTC GGT TCC TGG TGA TTT A	55	72	N/A
62	LIPC_RPO	AAC AGC CCC TAG TCA AGA GCA TGG CAC ACA ACA GAT GTT TCT AGA TTG GAT CTT GCT GGC AC	48	71	In lab
60	LCAT_LPO	GGG TTC CCT AAG GGT TGG AGA TGT GGT GAA CTG GAT GTG CTA CCG CAA GAC AGA GGA CTT	53	72	N/A
64	LCAT_RPO	CTT CAC CAT CTG GCT GGA TCT CAA CAT GTT CCT ACC CCT TGT CTA GAT TGG ATC TTG CTG GCA C	50	70	In lab
64	APOA4_LPO	GGG TTC CCT AAG GGT TGG AGG CGA GTG GTA TAC AAG CAG ACA AAG TCT TGC CGT GTA AAT GCC A	52	72	N/A
68	APOA4_RPO	AAT GTA ACG TGG CCT CCT TGT GCC CTT CCC CAC AGT GCC CTC TTC TCT AGA TTG GAT CTT GCT GGC AC	54	73	In lab
66	LPL_LPO	GGG TTC CCT AAG GGT TGG ACA AAA TAG CAG ATG TCA CTG AAG GAG AGC TCA GCG AGG GAG TGA TTG	52	71	N/A
70	LPL_RPO	/5Phos/ATT AAT AGC TGT ATT GAA AGG TGG GAG TCA GGT ACG GGG GAA GAG CGT CTA GAT TGG ATC TTG CTG GCA C	49	71	IDT
68	LIPG_LPO	GGG TTC CCT AAG GGT TGG AGA AAT GCC CAT GTA TGT GGA GCT AAG TGA GAC AGA GGG GTT GTC ATG CT	51	72	N/A
72	LIPG_RPO	TCA CTA TCC CCT TGT CCC ATG CTG CAA TCC GTT ATT TCA GAC GTG AGG ATC TAG ATT GGA TCT TGC TGG CAC	49	71	In lab
70	LDLR_LPO	GGG TTC CCT AAG GGT TGG AGG CTT ACG TAC GAG ATG CAA GCA CTT AGG TGG CGG ATA GAC ACA GAC TAT A	51	71	N/A

74	LDLR_RPO	GAT CAC TCA AGC CAA GAT GAA CGC AGA AAA CTG GTT GTG ACT AGG AGG AGG TCT AGA TTG GAT CTT GCT GGC AC	49	71	In lab
74	CETP_LPO	GGG TTC CCT AAG GGT TGG ATC TCA CCA CCT CTG CTG GCA CTG GTT GTC TCT TGC ACA TGG CTC CTT ACA ATC AA	53	73	N/A
78	CETP_RPO	AAT CAC ATC ATG CAA GTA ACG AGG GGG TAC ACA CGT GGT TTC CAC AGC TTA GGT ATC TAG ATT GGA TCT TGC TGG CAC	47	71	In lab
76	APOA5_LPO	GGG TTC CCT AAG GGT TGG AGA GGA CGC CCG CTG CAG TCC CCA GAA TCA AAG GAT GAT GTG GCG CAT CTA TGT TTC T	55	74	N/A
80	APOA5_RPO	/5Phos/TTG GAG AGT GTT GTA GGT CTG GAT TTG TAT GGG CAA TGT GTT TGT GCT TCG TGC GTG TCT AGA TTG GAT CTT GCT GGC	48	72	IDT
78	APOB_LPO	GGG TTC CCT AAG GGT TGG AGA GCA AGG GTT CAC TGT TCC TGA AAT CAA GAC CAT CCT TGG GAC CAT GCC TGC CTT TGA	53	73	
82	APOB_RPO	/5Phos/AGT CAG TCT TCA GGC TCT TCA GAA AGC TAC CTT CCA GAC ACC TGA TTT TAT AGT CCC CCT CTA GAT TGG ATC TTG CTG	48	71	IDT
80	ABCA1_LPO	GGG TTC CCT AAG GGT TGG ATT TCC AGA ACT TGG CTC CAG TCT GGT TGC TCG CCA TGA AGC ACT TAC AGA TAA ACC TCA TC	50	72	N/A
84	ABCA1_RPO	TTG GGC CAG TGC TTC CAT TTA CTG TCT CCT TTT GGC TTG CTT ATC CTT CCT TCT GCC TTC TTC TAG ATT GGA TCT TGC TGG CAC	48	72	In lab
82	APOC2_LPO	GGG TTC CCT AAG GGT TGG ACT GCC GTA CTT CCT CAT CTC CTA CGT GTG GAT GAT GAT ATT GTG CCC TGT GCA TGT TCT TCG T	51	72	N/A
86	APOC2_RPO	CAC CAA AAG TGC CTC TCT CAT AGA GCA GGT GAG AAC TCA GTG AGG AGA TGC AGG GAC ATG AGG TCT AGA TTG GAT CTT	51	72	In lab

57	DEFB103_RPO (MRC)	/5Phos/CAG ATC GGC AAG TGC TCG ACG CGT GGC CGA AAA TTC TAG ATT GGA TCT TGC TGG CAC	54	72	IDT
43	DEFB103_LPO (MRC)	GGG TTC CCT AAG GGT TGG ACT CAG CTG CCT TCC AAA GGA GGA A	56	70	N/A
50	CCR5_LPO	GGG TTC CCT AAG GGT TGG ACA TTA CAC CTG CAG CTC TCA TTT TCC ATA CA	48	69	N/A
54	CCR5_RPO	/5Phos/GTC AGT ATC AAT TCT GGA AGA ATT TCC AGA CTC TAG ATT GGA TCT TGC TGG CAC	43	66	IDT
62	CCR5_d32_RPO	/5Phos/TTA AAG ATA GTC ATC TTG GGG CTG GTC CTG CCG CTG CTT TCT AGA TTG GAT CTT GCT GGC AC	50	71	IDT

Table S2: Accuracy of Copy Number Calls is Dependent on Method of Calling

Sample Name	Ref CNV	Sex	CCR5 Genotype	P300_DEFB103A Ratio	P300_DEFB103A Copies Raw	P300_DEFB103A Copies Round	P300_DEFB103A Copies Cluster	P139_DEFB103A_1 Ratio	P139_DEFB103A_1 Raw	P139_DEFB103A_1 Copies Round	P139_DEFB103A_1 Copies Cluster
NA10861	3	F	WT/WT	0.81	3.24	3	3	0.75	3	3	3
NA07048	4	M	Δ32/WT	0.99	3.96	4	4	0.99	3.96	4	4
NA10846	5	M	WT/WT	1.23	4.92	5	5	1.25	5	5	5
HuRef	NA	M	WT/WT	1.21	4.84	5	5	1.21	4.84	5	5
LabRef1		F	Δ32/Δ32	1.22	4.88	5	5	1.29	5.16	5	5
LabRef5		F	WT/WT	1.41	5.64	6	6	1.56	6.24	6	6
232				0.81	3.24	3	3	0.73	2.92	3	3
233				1.01	4.04	4	4	1.01	4.04	4	4
226				1.02	4.08	4	4	0.95	3.8	4	4
**231**				**1.13**	**4.52**	**5**	**5**	**1.11**	**4.44**	**4**	**5**
228				1.15	4.6	5	5	1.14	4.56	5	5
**227**				**1.18**	**4.72**	**5**	**5**	**1.12**	**4.48**	**4**	**5**
**230**				**1.19**	**4.76**	**5**	**5**	**1.13**	**4.52**	**5**	**5**
225				1.2	4.8	5	5	1.28	5.12	5	5

Normalized ratios were generated against the 4 copy Coriell control sample. For each individual the raw copy number, as determined by multiplying the ratio by 4 (amount of copies in reference sample), is listed for each *DEFB103A* probe along with the cluster-determined copy number. Because converting raw copies to whole integers by rounding was determined to be inaccurate (raw copies for the 5-copy group did not cluster evenly around its whole integer value), k-means clustering was used to call copy number. Samples with previously typed CNV are indicated. For our sample set, *DEFB103A* had a range from 3-6 copies with the most common call being 5 copies. Sex: M=Male, F=Female; *CCR5* Genotype: Δ32/Δ32 = homozygous for delta 32 deletion, Δ32/WT = heterozygous for the Δ32 deletion, WT/WT = homozygous for the full length gene.
